# Well-being in the post-pandemic era: the role of meaning in life and resilience

**DOI:** 10.3389/fpsyg.2025.1696860

**Published:** 2026-01-13

**Authors:** Elif Baykal, Francoise Contreras, Ghulam Abid, Ignacio Aldeanueva-Fernández

**Affiliations:** 1 School of Business and Management Sciences, İstanbul Medipol University, Istanbul, Türkiye; 2 Research Center of Innovative Management, Azerbaijan State University of Economics, Baku, Azerbaijan; 3 School of Management and Business, Universidad del Rosario, Bogotá, Colombia; 4 Human Capital Division, Netsol Technologies, Lahore, Pakistan; 5 Department of Business Organization and Marketing, Universidad de Málaga, Málaga, Spain

**Keywords:** anxiety, life satisfaction, meaning in life, psychological resilience, well-being

## Abstract

**Introduction:**

The post-pandemic period significantly disrupted individuals’ daily routines, challenged their sense of purpose, and led to declines in psychological well-being. Prolonged uncertainty and loss of social continuity contributed to heightened anxiety for some individuals, while others continued to maintain life satisfaction. This study examines whether meaning in life enhances psychological resilience and whether resilience, in turn, influences anxiety and life satisfaction.

**Methods:**

Data were collected from 259 white-collar employees working in İstanbul. A quantitative, cross-sectional design was used to test the proposed relationships between meaning in life, resilience, anxiety, and life satisfaction through mediation analyses.

**Results:**

Meaning in life positively predicted resilience, and resilience positively predicted life satisfaction while negatively predicting anxiety. Meaning in life also exerted a direct positive effect on life satisfaction and a direct negative effect on anxiety. Mediation analyses revealed that resilience significantly mediated the effects of meaning in life on both anxiety and life satisfaction.

**Discussion:**

The findings highlight the protective role of meaning in life and resilience in mitigating anxiety and enhancing life satisfaction during the post-pandemic period. These results underscore the importance of developing interventions and organizational programs that cultivate meaning and strengthen resilience to support individual well-being in challenging contexts.

## 1 Introduction

The pandemic has led to an unprecedented worldwide disruption that changed many people’s routines, emotional health, and sense of meaning in life. Even in the post-pandemic period, many individuals continue to experience lingering psychological effects—including anxiety, uncertainty, and diminished well-being—while trying to reestablish normality in their personal and professional lives. This prolonged state of disruption provides a critical context for examining how internal resources such as meaning in life and resilience help individuals cope with ongoing and unexpected challenges. Although the psychological consequences of the COVID-19 pandemic have been widely documented (Abid et al., 2023; Ilyas et al., 2025), the processes through which individuals restore well-being and rediscover meaning in life in its aftermath remain insufficiently understood. This study aims to fill this gap by exploring how people reestablish purpose, coherence, and emotional balance in the post-pandemic period. As to previous researchers, in the coming years, the effect of the Covid-19 pandemic, coupled with social isolation and financial insecurity, is likely to affect people’s mental health (Sibley et al., 2020), and mental health is a global issue of concern (Ahmed et al., 2025; Richardson et al., 2021; Liu et al., 2024). As well-being and health are central to achieving sustainable development goals (SDGs), SDG 3 mandates “ensuring healthy lives and promoting well-being for all ages.” It poses an acute threat to the well-being of people owing to the challenges related to the social disruption it has triggered (Prime et al., 2020). This last pandemic has created an atmosphere of uncertainty wherein individuals are not sure about the proper way of reacting. Many people have dramatically changed their daily routines and restricted their interactions, resulting in lower levels of well-being (Lades et al., 2020; Milan et al., 2025). Disproportionate risk perceptions and emotion-driven behaviors have generated panic states and high levels of anxiety (Silahtaroglu et al., 2020), fear of failing in self-protection (Lin, 2021), and a sense of emptiness and meaninglessness (De Jong et al., 2020; Zeiler et al., 2025). Recently, Kjærum and Madsen (2025) and Leach et al. (2021) revealed the need for development approaches that can anticipate and respond to future uncertain shocks. Thus, the challenges of the post-pandemic era have been extensively studied in different contexts (Akram et al., 2022; Khor and Tan, 2023; Liu et al., 2023).

In these kinds of situations, having meaning in life helps make adverse events more bearable (De Jong et al., 2020; He et al., 2025). Coping with the pandemic has required mental resilience (Barzilay et al., 2020; Silveira et al., 2022). Similarly, in the post-pandemic world, it is essential to build worker resilience to navigate the changes in work and worker safety, health, and well-being (Peters et al., 2022). Higher meaning in life helps individuals to have a purpose in life. Those people having a meaning in life are more likely to make positive health-related lifestyle decisions (Schippers and Ziegler, 2019; Koga and Kubzansky, 2024) and alleviate the anxiety and panic reactions leading people to establish a stable basis to cope with the life challenges that this situation supposes (Trzebiński et al., 2020; Uzun and Arslan, 2025). Likewise, meaning in life leads people to use their resources to be more resilient and respond in the best way to this unusual adverse situation (Karataş and Tagay, 2021; Kagan and Ne’eman-Haviv, 2025), reducing the probability of experiencing depression, anxiety, and posttraumatic stress disorders (Feng et al., 2024; Lee et al., 2020; Schnell and Krampe, 2020). Moreover, according to Seligman (2018), meaning in life is one of the essential factors that make up well-being. A pandemic context can pose a serious threat to organizations’ vitality and survival; hence, organizations must help their workforce cope with and adjust to their newly altered work environment (Ashfaq et al., 2025; Carnevale and Hatak, 2020). Based on the related literature, this study assumes that meaning in life influences the capacity of resilience in the post-pandemic era. Likewise, it was predicted that the sense of meaning in life would positively affect life satisfaction in post-pandemic times, both directly and through resilience. It is assumed that it will reduce the anxiety levels of individuals.

This study suggests resilience as a mediator, considering that meaning in life can be reported to foster adaptive cognitive assessments, coherent thinking, and targeted behavior, thus encouraging the growth of resilience as a potential inner mental asset (Park, 2010). Previous studies has shown that having meaning in life improves resilience, consequently decreasing anxiety and increasing well-being (Arslan and Yıldırım, 2021; Karataş and Tagay, 2021). Some research studies consider resilience to be a stable characteristic that reduces anxiety (Connor and Davidson, 2003), whereas contemporary theories progressively express resilience as an unpredictable, flexible process affected by individuals’ capacity for creating meaning, especially during aftermath of adverse circumstances (Masten, 2014). This theoretical and temporal structure—that is, meaning → resilience → well-being—positions resilience as a mediator, more properly conveying its purpose as a basic psychological system that explains the causal connection among meaning in life and enhanced psychological outcomes.

In the related literature, there is a considerable amount of proof claiming that meaning in life improves individuals’ resilience and well-being; however, there is a shortage of studies investigating how it helps individuals to cope with the difficulties caused by the pandemic. On the one hand, we came across a satisfactory amount of proof demonstrating that retaining a feeling of meaning in life enhances psychological resilience and well-being; yet, there is a shortage of investigations looking at its function in allowing people to deal with the obstacles brought about by the global pandemic. Furthermore, the constantly shifting processes whereby meaning in life reduces anxiety and encourages proactive actions in the face of novel adverse events remain inadequately researched. In other words, it is seen that in spite of the extensive empirical studies on the psychological consequences of Covid-19, there is merely a limited quantity of knowledge regarding the way meaning in life supports individual-level psychological resilience and the methods of emotional adjustment. Furthermore, previous studies rarely investigate this link within different cultures and high-risk environments. Moreover, the path through which meaning in life decreases anxiety levels and boosts adaptation—especially via psychological resilience—remains insufficiently examined; hence, we wanted to trace this association in this particular study. Lastly, extending the scope of our study to a new cultural and occupational context enhances the originality and theoretical value of our study and helps clarify the novel insights our model introduces. Hence, with the related field study, we wanted to test the assumed positive effect of meaning in life on resilience and the mediator effect of resilience in the relationship between meaning in life and life satisfaction and anxiety levels of individuals in the aftermath of the pandemic. This research is the first empirical study to show the positive effect of a sense of meaning in life on psychological resilience in the post-pandemic period in the Turkish context.

## 2 Literature review and hypotheses development

This research is framed within the Shattered Assumptions Theory (SAT), which proposes that a traumatic experience can change both victims’ and survivors’ self- and world views (Janoff-Bulman, 1992). According to this theory, adverse events affect basic assumptions of the individuals, such as benevolence, meaningfulness, and self-worth. These beliefs constitute the individuals’ conceptual system, allowing them to recognize, plan, and act. However, traumatic life events such as a pandemic can shatter these fundamental assumptions. Unexpected situations like pandemics create a challenge for better understanding and using tools for analysis and managing risks (Munasinghe, 2020) and create an occasion for questioning the meaning in life. Thus, in this research, it is proposed that meaning in life (a basic assumption) has a positive or negative influence on people’s well-being (life satisfaction and anxiety, respectively) directly or mediated by resilience.

### 2.1 Meaning in life and life satisfaction

For years, spirituality and finding the meaning in life have been considered basic phenomena in the context of human existence (Skrzypińska, 2021). Despite the many ambiguities shrouding the experience of life as meaningful, the things that make life meaningful are resources that are widely available to most people, such as engaging in social relationships and having religious faith (King and Hicks, 2021). Thus, meaning in life is understood as a cognitive and emotional evaluation of one’s life. According to Steptoe and Fancourt (2020), meaning could be considered a complex construct that encompasses the feeling that life makes sense and is valuable, leading to establishing a direction in life; or, in other words, meaning in life can be understood as the presence of meaning and how intensely people seek life meaning (Li et al., 2021). According to Martela and Steger (2016), meaning in life involves coherence, purpose, and significance. Meaningfulness and purposefulness in life can be considered the main components of eudaimonic well-being (Steptoe and Fancourt, 2020); these are related to life satisfaction (Compton, 2000).

There is evidence that meaning in life contributes to increased life satisfaction and happiness while decreasing depression, anxiety, and other undesirable states related to negative affect (Williamson and Geldenhuys, 2014). There was evidence that naturally optimistic persons were more likely to be satisfied with their employment (Omar et al., 2019); for example, a recent study with Turkish retirees confirmed the positive effect of meaning in life on life satisfaction (Baytan and İşözen, 2019). Considering these findings, we hypothesized that:

H_1_: Meaning is life is positively related to life satisfaction.

### 2.2 Meaning in life and resilience

Meaning in life can be affected by the uncertainty that generates an adverse event like a pandemic. Uncertainty can boost meaninglessness by affecting purposiveness and confidence about the effect of one’s actions (Stillman and Baumeister, 2009). In front of a sudden, unexpected, and critical event like the last pandemic, people have had to use their best personal resources to adapt and cope in a better way; in other words, they should be resilient in a context of post-traumatic growth. Meaning in life also creates a particular sensitivity to recognize the situational cues of potential risks, enabling individuals to better cope with the situations (Hooker et al., 2018). Due to this, individuals who sustain a meaningful life can endure better and recover from traumas (Yang, 2020). Thus, a life with meaning leads to greater tolerance to cope with difficulties and contribute to greater resilience (Martela and Steger, 2016). Because of that, meaning in life may be considered as a facilitator to cope successfully with adverse events (Karataş and Tagay, 2021) and, according to Ward et al. (2023), has been proposed to improve resilience, becoming a feature that acts as a protective factor that helps to preserve well-being (Blackburn and Owens, 2015). Resilience is not a rare characteristic, and most individuals are resilient to some extent (Prayag et al., 2020). However, the more resilient an individual is, the more able they are to successfully cope, adapt, and thrive in challenging times (Chen and Bonanno, 2020).

Resilient individuals tend to perceive more personal control and social support, are more optimistic and confident, have a greater sense of purpose (Prayag et al., 2020), and have a moral compass and use cognitive flexibility (Luo et al., 2022). The sense of purpose related to meaning in life helps people reduce the stress produced by adverse events and the experience of individual and social growth, increasing their resilience capacity (Ryff, 2014). Actually, people should regain their sense of meaning in life to be resilient. Individuals with low levels of meaning in life forego struggling when they face a difficult situation (Theron and Theron, 2014), so it is necessary to identify individuals at risk for chronic distress in order to build resilience (Lin et al., 2022). Following the assertions of Hogan et al. (2020), some recent studies have found that during the last pandemic, meaning in life has had an effect on resilience (Akyıl et al., 2025; Arslan and Yıldırım, 2021; Kagan and Ne’eman-Haviv, 2025; Karataş and Tagay, 2021; Yıldırım et al., 2022; Konstatopoulou, 2024). Supported in this literature, we posit the following hypothesis:

H_2_: Meaning in life is positively related to resilience.

### 2.3 Resilience and life satisfaction

Resilience should be considered as a dynamic process enabling individuals to achieve a favorable outcome in the face of adversities such as a pandemic situation (Chen and Bonanno, 2020). Under normal conditions, the absence of balance and resilience in life gives way to behavioral and emotional problems such as anxiety, guilt, melancholy, and lower productivity levels (Prithi and Vasumathi, 2020). Resilience contributes to business continuity and sustainability (Jain et al., 2020). In contrast, it contributes to a more positive mood and life satisfaction. Thus, it has been asserted that resilience contributes to greater life satisfaction (Cohn et al., 2009; Miri et al., 2021; Baykal, 2023). Empirical studies have provided evidence about the positive effect of resilience on life satisfaction (Arslan, 2019; Paul et al., 2019). Similarly, during the pandemic, Hadjicharalambous et al. (2021) demonstrated the positive effect of resilience on increasing life satisfaction. In the same line, Anderson et al. (2020) confirmed the positive relationship between resilience and quality of life, related to life satisfaction. According to the findings of a study done by Pathak and Joshi (2021), those who have strong resilience report more satisfaction with the overall quality of their lives. Recently, other studies published in times of pandemic and post-pandemic also confirmed the positive relationship between resilience and life satisfaction (Barzilay et al., 2020; Baykal, 2020; Gündoğan, 2021; Haider et al., 2022; Kütük et al., 2023; Ran et al., 2020; Tamarit et al., 2023; Kan et al., 2025). Resilience has been confirmed to promote adjustment in individuals’ mental health. Supported by these findings, we posit the following hypothesis:

H_3_: Resilience is positively related to life satisfaction.

### 2.4 Meaning in life and anxiety

Fear of death crushes individuals’ fundamental assumptions about the world and the self (Chang et al., 2021) and leads to questioning meaning in life. In some way, meaning helps individuals keep stability, creates a higher awareness about the current moment, and gives cues to identify potential threats (Kelso et al., 2020). Meaning in life helps buffer the adverse psychological outcomes that may occur after a traumatic event. Under adverse conditions, excessive anxiety can affect individuals’ physical and mental health, involving mood disorders, and in some cases can lead to psychiatric disorders (Lawrence et al., 2019). In this regard, there is evidence about the negative effect of the pandemic on general health and life satisfaction in different latitudes (Özmen et al., 2021; Rogowska et al., 2020). In times of pandemic and post-pandemic, health problems related to anxiety are increasing, as are the requirements of interventions and medications to treat it. There is evidence about the effect of meaning in life on decreasing individuals’ anxiety in studies published before, during, and after the recent pandemic (Arslan et al., 2020; Carreño et al., 2020; Milman et al., 2022; Seidel et al., 2023; Seidel-Koulaxis and McNally, 2024; Trzebiński et al., 2020). According to the above, we raise the following hypothesis:

H_4_: Meaning in life is negatively related to anxiety.

### 2.5 Resilience and anxiety

Pandemics can heighten the stress levels of individuals, creating anxiety as a usual response (Roy et al., 2020). Under adverse conditions, anxiety can become a chronic state resulting from continuous stress, increasing concerns about uncontrollable situations (Poudel-Tandukar et al., 2019). While resilience enables individuals to cope with problems in life, anxiety makes individuals more vulnerable in adverse situations (Zaleski et al., 2019). There is evidence about the inverse effect of resilience on anxiety levels (Hjemdal et al., 2011; Ko and Chang, 2019; Liu et al., 2025). This impact was also confirmed in employees (Shin et al., 2019) and patients with depression (Min et al., 2015). In this line, Poudel-Tandukar et al. (2019) found that higher resilience is associated with reduced anxiety and depression. Also during pandemic and post-pandemic times there is evidence about the negative effect of resilience on anxiety (Barzilay et al., 2020; Lyu et al., 2022; Rayani et al., 2022; Song et al., 2021; Traunmüller et al., 2023). Based on the above findings, it is hypothesized that:

H_5_: Resilience is negatively related to anxiety.

### 2.6 Resilience as a mediator

The Shattered Assumptions Theory (1992) was helpful in creating our mediation model. This theory posits that traumatic events, like the post-pandemic period, may destroy people’s basic assumptions about the world as valuable, foreseeable and benign. Whenever individuals restore meaning following a major change, they gain back a feeling of unity and purpose, that is a vital component of mental recovery making people more resilient since individuals who acquire meaning a second time may reorganize their thoughts, view problems in an entirely different perspective, and recapture a sense of having control over their own lives. When resilience increases, it acts like a conduit that minimizes vulnerability to anxiety and promotes enhanced performance and increased life satisfaction. Therefore, based on Shattered Assumptions Theory, meaning in life is not something that occurs along with resilience; it actually drives the process of rebuilding that leads to resilience, which then affects mental health outcomes. This theoretical sequence corresponds directly with the proposed mediation pathways in the present study.

Klutsey and Mahama (2025) study revealing the statistically significant impact of academic resilience as a mediator in the relationship between meaning in life and life satisfaction and Chen et al. (2024) study revealing the mediator effect of resilience in the relationship between meaning in life and happiness were illuminating for us. Although we did not find studies where psychological resilience mediated the relationship between meaning in life and life satisfaction and anxiety, there is evidence about the mediating role of resilience on other psychological well-being variables. For instance, Faircloth (2017) revealed the effect of resilience as a mediator in the relationship between adverse life events and psychological well-being. Ika et al. (2021) found the same effect between spirituality and quality of life. The findings show that resilience plays a role in mediating the relationship between social support and life happiness (Cao and Zhou, 2021).

On the one hand, there are also significant studies revealing the mediator effect of resilience in the relationship between meaning in life and lower levels of anxiety, such as Akdağ et al. (2024) study revealing that psychological resilience acts as a mediator in the relationship between meaning in life and psychological distress in adolescents and Prisăcaru (2025) revealing the mediating role of psychological resilience and coping mechanisms in the relationship between personality traits and stress, anxiety, and depression. As seen in the example, since meaning in life is associated with resilience, and both resilience and meaning in life are predictors of life satisfaction, it is necessary to investigate the possible mediating effects of resilience on the relationship between meaning and life satisfaction to further our knowledge about the mental health of individuals. Supported by these findings, we assumed that meaning in life would lead to greater life satisfaction and lower anxiety levels when individuals are more resilient. Thus:

H_6a_: Resilience mediates the relationship between meaning and life satisfaction.

H_6b_: Resilience mediates the relationship between meaning and anxiety.

**Figure 1:**
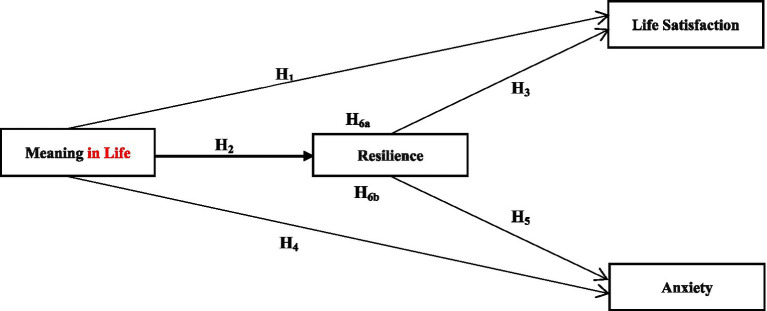
Conceptual proposed model and hypotheses.

In support of the presented literature and discussion, we hypothesize that meaning in life affects individuals’ well-being (higher life satisfaction, lower anxiety) either directly or through resilience. The hypothesized model is presented in Figure 1.

## 3 Methodology

### 3.1 Sample and procedures

In the present study, we have adopted a convenience sampling strategy to recruit participants from the service sector due to the unavailability of a known population. This choice was driven by the practical considerations, including the confidentiality restrictions related to the list of employees and their roles, as well as the access permissions for employees and the organization. The organizational research is not suitable due to the unavailability of a sampling frame, and organizations restrict direct access to personal records. Hence, convenience sampling with voluntary participation served as the most ethically appropriate technique. Using this technique, we can easily collect data from individuals who are willing to participate, easily accessible, and able to offer informed responses.

To ensure that participants provide reliable responses, we consider only full-time employees who have a formal organizational role, have been in their current organization for at least 6 months, and are able to read and comprehend the questionnaire items independently. We have excluded interns, part-time employees, contractual employees, and employees who may be transitioning from their current role/team. Applying these criteria strengthened the internal validity of the study.

Data were collected from companies in the service sector located in Istanbul with more than 1,000 employees. Istanbul is the country’s primary business center, and nearly all firms are in the service sector. As the research was conducted in Turkey, all survey constructs developed in English were translated into Turkish using a rigorous, multi-step forward-backward translation method by Brislin (1970) to ensure semantic, conceptual, and cultural equivalence. Two bilingual subject-matter experts independently translated the scale items into Turkish. After that, an independent pair of experts performed a back-translation into English. All discrepancies found during any stage were reviewed and resolved through consensus discussions. This process ensures that the Turkish version retains the psychometric integrity, linguistic clarity, and theoretical meaning of the original scales. Informed consent was obtained. A total of 959 surveys were sent to the companies to be applied by the employees. Employees filled out a total of 259 questionnaires. The entire data collection process was done between April 2023 and February 2024. Participants were primarily young employees; 53% were under 40 years old, 27% were between 40 and 50 years old, and 20% were older than 50 years old. Most of the participants were male (67%). Regarding education, slightly more than half of the sample (51%) hold a university degree, and 27.7% have obtained a master’s degree. 13% of the participants have a PhD degree.

### 3.2 Measures

The questionnaires were sent to the target sample through an online platform. Replies to survey items were collected using a five-point Likert-type scale, ranging from “strongly disagree” (1) to “strongly agree” (5). We also included some reverse items. Meaning in life was measured by a 3-item subscale of the broader construct of well-being developed by Peterman et al. (2002). Despite its brevity, this validated short form has been widely used in survey-based studies and is considered appropriate when respondent burden is a concern. A sample item is ‘*I have a reason for living*’. The Cronbach’s *α* scale for this is 0.75. We also used the 6-item Psychological Capital Scale, designed by Luthans et al. (2007), to measure resilience. A sample item is ‘*I usually manage difficulties one way or another at work*’. The Cronbach’s α scale for this is 0.77. Life satisfaction was measured using the 5-item Life Satisfaction Scale developed by Dağlı and Baysal (2016). A sample item is ‘*I am living a life close to my ideals’.* The Cronbach’s α scale for this is 0.86. Lastly, for measuring anxiety, we used the 5 items scale designed by Zaleski et al. (2019). A sample item is ‘*I’m afraid my life will get worse in the future*’. The Cronbach’s α scale for this is 0.83.

## 4 Data analysis

### 4.1 Analysis of construct validity

Confirmatory factor analysis (CFA) was performed to investigate the construct’s validity utilizing the Fornell and Larcker (1981) validity assessment criterion. Before measuring discriminant and convergent validity, model fit indices were assessed for the proposed measurement model along with other substitute models. At first, we examined the four-factor measurement model. For this, we drew all items related to our four constructs (meaning, resilience, life satisfaction, and anxiety) in AMOS, then allowed them to relate freely to their respective variables. The output of our four-factor model was depicting a good fit (see Table 1) as Tucker-Lewis Index (TLI) = 0.91, Normed Chi-square (χ^2^/df) = 2.25, Confirmatory Fit Index (CFI) = 0.93, Incremental Fit Index (IFI) = 0.93, Goodness of Fit Index (GFI) = 0.90, Standardized Root Mean Square Residual (SRMR) = 0.07, and Root Mean Square Error of Approximation (RMSEA) = 0.07. The result of the above indices falls into the acceptable limit: χ^2^/df < 3, TLI > 0.90, IFI > 0.90, CFI > 0.90, GFI > 0.80, RMSEA < 0.08, and SRMR < 0.05 (Kline, 2023). Moreover, the 4-factor measurement model was also compared with the other three alternate models to see the best fit model for our collected data set. It is demonstrated that our 4-factor model best fits the data set, and none of the alternative models provided an acceptable fit. Thus, the result supports the idea that meaning, resilience, life satisfaction, and anxiety are distinct constructs.

**Table 1:** Fit indices for models comparison.

Models	χ^2^	χ^2^/df	SRMR	RMSEA	GFI	CFI	TLI	IFI
Measurement Model	249.16	2.25	0.07	0.07	0.90	0.93	0.91	0.93
3 Factor Model^a^	329.50	2.89	0.07	0.09	0.87	0.88	0.86	0.88
2 Factor Model^b^	597.36	5.06	0.08	0.13	0.75	0.74	0.70	0.74
1 Factor Model^c^	708.62	5.96	0.09	0.14	0.71	0.68	0.63	0.68

*n* = 259, Full measurement model is compared with alternate models. 3 Factor Model^a^: Life satisfaction and meaning combined into one factor. 2 Factor Model^b^: Life satisfaction and meaning combined into one factor, resilience and anxiety combined into another factor. 1 Factor Model^c^: All variables combined into one factor.

Likewise, the validity of the constructs was examined through convergent and discriminant validity. Composite reliability of the study variables and factor loadings of items are needed to examine the convergent validity (Hair et al., 2014). The values of factor loading for each item should be greater than 0.40, and composite reliability should be greater than 0.70 (Fornell and Larcker, 1981; Hair et al., 2014). The findings are presented in Table 2. The results indicated that the loadings for all items were greater than 0.40. Furthermore, all variables have CR greater than 0.70. Hence, satisfying the conditions of convergent validity.

**Table 2:** Reliability and validity of the study variables.

Study variables	Convergent validity		Discriminant validity			
	Factor loadings	Composite reliability	1	2	3	4
1. Anxiety	0.717, 0.686, 0.801, 0.752	0.829	**0.740**			
2. Life Satisfaction	0.659, 0.828, 0.726, 0.775, 0.727	0.861	−0.579	**0.745**		
3. Resilience	0.476, 0.535, 0.836, 0.622, 0.678	0.771	−0.412	0.543	**0.642**	
4. Meaning	0.750, 0.687, 0.696	0.755	−0.567	0.669	0.549	**0.712**

Values in diagonal represent the squared root estimate of the average variance extracted. Bold values in diagonal represent the squared root estimate of the average variance extracted.

Finally, the Fornell and Larcker (1981) method was used to ensure the discriminant validity. According to them, the square root of the AVE of every study variable should be greater than the correlations of the other constructs. All diagonal values were greater than inter-construct correlation (e.g., for anxiety, square root of 0.740 > −0.579, −0.412, −0.567, and for meaning, square root of 0.712 > 0.549, 0.669, −0.567, etc.). Therefore, convergent and discriminant validities are supported (see Table 3).

**Table 3:** Descriptives and correlations.

Variables	Mean	SD	1	2	3	4	5	6	7
1. Age	-	-	1						
2. Gender	-	-	0.28^**^	1					
3. Education	3.51	0.90	0.05	−0.04	1				
4. Meaning in Life	4.20	0.72	0.20^**^	0.13^*^	0.12	**(0.75)**			
5. Resilience	3.89	0.60	0.19^**^	0.14^*^	0.03	0.48^**^	**(0.77)**		
6. Life Satisfaction	3.44	0.77	0.14^*^	0.08	0.16^**^	0.54^**^	0.47^**^	**(0.86)**	
7. Anxiety	2.34	0.93	−0.13^*^	−0.11	−0.06	−0.45^**^	−0.36^**^	−0.49^**^	**(0.83)**

*n* = 259; Bold values in diagonal represent Cronbach’s α. **Correlation is significant at the level of 0.01 (**) and 0.05 (*).

### 4.2 Multicollinearity statistics

We examined the multicollinearity between meaning, resilience, and life satisfaction using the variance inflation. We examined the multicollinearity between meaning, resilience, and life satisfaction using the variance inflation factor (VIF) and tolerance index. The VIF value should not exceed 10, and the tolerance value should be larger than 0.10 (Pallant, 2011). The VIF values for meaning (1.56), resilience (1.42), and life satisfaction (1.53) were all less than 10, according to the findings of this study. Furthermore, meaning, resilience, and life satisfaction had tolerance values greater than 0.10, ranging from 0.64 to 0.71. None of these indicators showed an issue with multicollinearity in our investigation.

### 4.3 Descriptives and correlations

Before testing the proposed relationships among study variables, bivariate correlation analyses were performed. Table 1 shows the standard deviations (SD), means, alpha reliabilities, and correlations. In support of the proposed hypotheses, the results reveal that meaning in life is positively and significantly associated with life satisfaction (*r* = 0.54, *p* < 0.01) and resilience (*r* = 0.48, *p* < 0.01), which are in accordance with our H1 and H2. It is found that anxiety is negatively and significantly associated with meaning (*r* = −0.45, *p* < 0.01) and resilience (*r* = −0.36, *p* < 0.01). These outcomes are in accordance with H4 and H5, respectively. Finally, the result shows that resilience is positively associated with life satisfaction (*r* = 0.47, *p* < 0.01), supporting H3. All correlation values fall within the range of ±0.30 to ±0.70, indicating a moderate level of association among the study variables.

## 5 Results

We analyze whether the influence of meaning on life satisfaction and anxiety could be explained through resilience by employing PROCESS macro (model 4, Preacher and Hayes, 2008). A 5000 bootstrap resampling was performed. The outcomes of PROCESS for life satisfaction (Table 4) illustrate that the influence of meaning in life on resilience was significant and positive (*β* = 0.40, *t* = 8.85, *p* < 0.00), providing support for Hypothesis 2. Further, the impact of meaning in life on life satisfaction was significant and positive (*β* = 0.43, *t* = 7.07, *p* < 0.00). Additionally, resilience positively influenced life satisfaction (*β* = 0.35, *t* = 4.65, p < 0.00). These results offer support for Hypothesis 1 and 3, respectively. The mediation analysis results were also observed using the Sobel test. The formal two-tailed significance test demonstrates that the indirect impact (0.14) is significant, with Sobel z = 4.10, *p* < 0.00. The bootstrapping also confirmed the result of the Sobel test (see Table 4) with a similar indirect effect value of 0.14. The 95% bootstrap confidence interval for this indirect effect did not contain zero (0.07, 0.23), supporting Hypothesis 6a.

**Table 4:** Results of simple mediation model for life satisfaction.

Direct effect model						
Predictor		Outcome = Resilience				
		Β	SE	T		*p*
Meaning in Life		0.40	0.05	8.85	0.00	
Constant		2.21	0.19	11.44	0.00	
Predictor		Outcome = Life Satisfaction				
Meaning in Life		0.43	0.06	7.07	0.00	
Resilience		0.35	0.07	4.65	0.00	
Constant		0.27	0.28	0.96	0.34	
Total effect model						
Predictor		Outcome = Life Satisfaction				
Meaning in life		0.57	0.06	10.25	0.00	
Indirect effect and significance using the normal distribution						
	Value	SE	LL 90% CI	UL 90% CI	Z	p
Sobel	0.14	0.04	0.07	0.23	4.10	0.00
Bootstrap results for indirect effect of X on Y						
		Resilience	SE	LL 90% CI	UL 90% CI	
Effect		0.14	0.03	0.07	0.23	

*n* = 259; β, Regression Coefficient; SE, Standard Error; Bootstrap Sample Size = 5,000; LL, Lower Limit; CI, Confidence Interval; UL, Upper Limit.

The outcomes of PROCESS results for anxiety (Table 5) illustrate that the effect of meaning in life on resilience was significant and positive (*β* = 0.40, *t* = 8.85, *p* < 0.00), providing support for Hypothesis 2. Further, the impact of meaning in life on anxiety was significant and negative (*β* = −0.46, *t* = −5.71, *p* < 0.00). Finally, resilience negatively influenced on anxiety (*β* = −0.29, *t* = −2.97, *p* < 0.00). These provide support for Hypothesis 4 and Hypothesis 5. The result of the mediation analysis was also tested using the Sobel test. The formal two-tailed significance test demonstrates that the indirect impact (−0.12) is significant with Sobel z = −2.80, *p* < 0.01. The bootstrapping also confirmed the Sobel test (Table 5) with a similar indirect effect value −0.12. The 95% bootstrap confidence interval for this indirect effect did not contain zero (−0.23, −0.02), supporting Hypothesis 6b.

**Table 5:** Results of simple mediation model for anxiety.

Direct effect model						
Predictor		Outcome = Resilience				
		Β	SE	T		*p*
Meaning in life		0.40	0.05	8.85	0.00	
Constant		2.21	0.19	11.44	0.00	
Predictor		Outcome = Anxiety				
Meaning in life		−0.46	0.08	−5.71	0.00	
Resilience		−0.29	0.10	−2.97	0.00	
Constant		5.40	0.37	14.60	0.00	
Total effect model						
Predictor		Outcome = Anxiety				
Meaning in life		−0.58	0.07	−8.04	0.00	
Indirect effect and significance using the normal distribution						
	Value	SE	LL 90% CI	UL 90% CI	Z	p
Sobel	−0.12	0.05	−0.23	−0.02	−2.80	0.01
Bootstrap results for indirect effect of X on Y						
		Resilience	SE	LL 90% CI	UL 90% CI	
Effect		−0.12	0.05	−0.23	−0.02	

## 6 Discussion

In the post-pandemic era, this study aims to provide evidence about the variables that can help keep or improve people’s well-being (assessed by life satisfaction), diminishing the psychological discomfort (evaluated by anxiety states). Our findings support the assertion that meaning in life is an excellent source of well-being as far as helping people establish the basis to cope with adverse situations (De Jong et al., 2020). In general terms, and line with the previous studies (Silahtaroglu et al., 2020; Trzebiński et al., 2020; Williamson and Geldenhuys, 2014), we can affirm that meaning in life influences positively life satisfaction while diminishing levels of anxiety. Our findings align with the research conducted by Baytan and İşözen (2019), who found that meaning in life was related to life satisfaction, in Turkish retirees, Zhang et al.’s (2025) study revealing the relationship between presence of meaning in life and life satisfaction among Chinese young adults and Kwok et al. (2025) study showing the impact of *Stability and changes in meaning in life profiles and their impact on mental health among Chinese university students* So, in support of the previous literature, our results allow us to accept the H1, indicating that in the post-pandemic years, individuals who experience more meaning in their life tend to perceive greater life satisfaction.

On the other hand, we found that meaning in life influences peoples’ resilience, supporting the H2. This finding is coherent with past studies like Karataş and Tagay (2021), Kagan and Ne’eman-Haviv (2025) and Karimi Dastaki and Mahmudi (2024) revealing the positive effect of meaning in life on psychological resilience levels of individuals. Likewise, as hypothesized, resilience influences life satisfaction in the post-pandemic period, supporting the H3. This result is supported in previous literature (Haider et al., 2022; Kütük et al., 2023; Tamarit et al., 2023). This finding is very relevant because resilience is a capacity that can be developed, and its role is crucial to get over different adversities a better way (Chen and Bonanno, 2020). Contributing to people’s well-being, in this study, we found that meaning in life had an inverse relationship with anxiety, supporting the H4. This result confirms that meaning in life, as a resource, helps individuals keep stability in adverse situations, reducing the anxiety suggested by researchers from past studies (Uzun and Arslan, 2025; Yıldırım et al., 2025). Regarding the adverse effect of anxiety on peoples’ health and well-being, meaning in life could be an essential resource that has to be encouraged in different life spheres, being the work context one of the most important (Seidel et al., 2023). The results of this study can be helpful for this purpose.

Likewise, this research provides evidence about the effect of resilience in reducing anxiety in the post-pandemic world, giving support to H5. Our results are similar to those obtained by other authors (Lyu et al., 2022; Traunmüller et al., 2023). This effect is crucial because anxiety makes people more vulnerable in adverse situations (Zaleski et al., 2019; Riskind, 2024) and is in line with the other authors’ findings (Milman et al., 2022; Prisăcaru, 2025). Lastly, our findings confirm the critical mediator role of resilience between different variables related to people’s well-being, as found by other authors (Ika et al., 2021; Roohi et al., 2025; Sarrionandia et al., 2018). This study adds new evidence about the other mediator role of resilience related to people’s well-being. In this study, resilience was demonstrated to be a valid mediator of people’s well-being in both the positive effect of meaning in life on life satisfaction and in the inverse effect of meaning in life on anxiety, accepting H6a and H6b, respectively. Currently, no other study tests the exact same mediator models as ours; however, some studies examine similar dimensions. For instance; our results show similarity with the study of Du et al. (2017) showing the moderator effect of resilience in the relationship between meaning in life and psychological well-being and Klutsey and Mahama (2025)‘s study revealing the effect of resilience as a mediator between meaning in life and subjective happiness and Brudek and Sękowski (2021) study that revealed the same relationship with the mediator effect of wisdom. Moreover, our findings in H6b is confirming the results of Akdağ et al. (2024) study revealing the mediator effect of psychological resilience in the relationship between meaning in life and psychological distress in adolescents, Pérez-Aranda et al. (2021) study that examined the impact of mindfulness and self-compassion on anxiety and depression and revealed the mediating role of resilience in this relationship and Ma et al. (2025) study revealing the mediating role of presence of meaning in life in the relationship between self-compassion and psychopathological symptoms Hence, it can be claimed that this study contributes to the literature in the point that it is the first model attempting to explain effect of meaning in life on anxiety and life satisfaction with the mediating effect of resilience.

In this study, we have figured out that our findings matched with Self-Determination Theory (SDT) that provides an explanatory framework, suggesting that individuals feel greater life satisfaction, incase their psychological needs are satisfied (Ryan and Deci, 2000). In this point, Meaning in life acts as an intrinsic psychological resource enabling individuals to interpret their experiences in coherent ways through which they facilitate need fulfillment and augmenting overall life satisfaction. Our findings are also parallel with assumptions of Existential Positive Psychology approach, which suggests that individuals gain resilience and psychological well-being by extracting meaning from challenges and adversity. According to this approach, meaning in life mitigates adverse psychological conditions and fosters adaptive functioning by allowing individuals to contextualize challenges within a meaningful narrative. Lastly, the results can also be understood in terms of an stress-appraisal framework. This framework says that people who have a strong sense of meaning see stressful events as less dangerous, which means they feel less anxious and happier. These complementary theories collectively offer a cohesive explanation for the pathways identified in our study, illustrating that meaning in life is not only linked to well-being outcomes but also serves as a theoretically substantiated mechanism that influences how individuals interpret experiences, navigate psychological stress, and ultimately report enhanced life satisfaction.

## 7 Theoretical and managerial implications

In times of post-pandemic, meaning in life and resilience are relevant concepts that need to be encouraged to preserve people’s health and well-being during adversity. This assertion is confirmed with our findings. This study shows the effect of sense of meaning in life on psychological resilience, life satisfaction and anxiety level with a highly explanatory model and proves the mediating effect of psychological resilience in this network of relationships. As the first model studied in the literature, it fills an important theoretical gap. Thanks to this study, it has been underlined how important the sense of meaning is in adverse times such as post-pandemic era, and the effect of resilience on individuals’ life satisfaction and anxiety has been proven.

Under pandemic times and beyond, the work environment takes on a particular relevance and responsibility to strengthen the meaning in life in the employees, promoting their well-being. As a result, employees can strengthen their resilience capacity, making them more able to successfully cope and thrive at difficult times now and in the future with other unforeseen adversities. Findings of this study are valuable outputs for companies that, from management practices and human resources management, can promote the well-being of the employees. In this regard, reducing uncertainty can encourage meaning in life and the boost confidence of employees to cope with the post-pandemic years. Likewise, companies can contribute to greater resilience by providing support for meaning in life, allowing employees to focus on the parts of the work that brings joy and meaning, thus stimulating resilience. Based on our results, it can be figured out that finding meaning in life can act as an essential strategic tool. Through ensuring that HR initiatives adequately clarify the societal significance of every employee’s role, assigning roles to each individual’s competencies, and offering employees opportunities to shape their own jobs and feel empowered, organizations may assist employees realize that their work has meaning. Also, there need to be mentorship programs, feedback mechanisms, and training programs which illustrate employees the way their work fits into the wider scheme of the company.

## 8 Conclusion

This study is illuminating about the effects of meaning in life during the post-pandemic times. As it is revealed in the analysis results meaning has a positive effect on resilience and resilience has a positive effect on life satisfaction and an inverse effect on anxiety levels of individuals. In this study, the mediator role of resilience has been revealed in the relationship between meaning in life and life satisfaction and anxiety levels of individuals. In other words, resilience has been confirmed as a valid mediator of people’s well-being through its effect on life satisfaction and through its inverse on anxiety. The model is important in terms of proving how the sense of meaning experienced by individuals in general is effective in overcoming difficult processes and making sense of its positive effect on increasing well-beings of individuals.

In further studies, whether the sense of meaning affects other processes related to stress or not can be examined making it a more elucidatory study. Furthermore, the model in this study can be situated in the context after the covid-19 outbreak. Actually, this study is limited to Turkey business context and specific to service sector. The hypothesized relationships can be tested in different countries and cultures too. In later studies, the effects of meaning in life can be examined on psychological resources other than resilience in struggling with crisis periods such as pandemics.

### 8.1 Limitations and further studies

Without doubt, our study has some limitations that should be acknowledged to guide the interpretation of the findings. The cross-sectional design and convenience sampling technique restrict the ability to generalize the conclusions about causality among the variables. Longitudinal or experimental designs could have allowed further researchers to test the model more rigorously. On the other hand, our study relied entirely on self-report measures, which can create biases. In data analysis, using mixed-methods could have enriched the robustness of our findings. Moreover, the properties of our sample may have limited generalizability of the study; the demographic composition could have developed. Future research should replicate the study in more diverse samples across different age groups, cultures, and socioeconomic backgrounds to enhance external validity. Finally, the analytical approach focused on specific mediators and outcomes, which means alternative mechanisms such as coping strategies, emotion regulation, or social connectedness were not included and may play important explanatory roles.

Future studies could use longitudinal studies covering a long time period or cross-lagged panel designs. On the other hand, further empirical studies covering psychological interventions like compassion-focused interventions or goal-setting workshops can boost meaning or promote resilience in our modal. Cross-cultural studies comparing our models in different cultures can also be enlightening to gain a deeper understanding. In addition, different mediators or moderators, such as perceived organizational support, ethical organizational climate, emotion regulation, and positive leadership models, will advance understanding of how meaning in life affects psychological well-being.

## Data Availability

The original contributions presented in the study are included in the article/supplementary material, further inquiries can be directed to the corresponding author.
